# History, Description and Statistics, of the Bloomingdale Asylum for the Insane; Report of the Pennsylvania Hospital for the Insane, for the Year 1847

**Published:** 1848-07

**Authors:** 


*History, Description and Statistics, of the Bloomingdale Asylum for the Insane.* By Pliny Earle, M. D., Physician to the Institution, &c. New York, 1848.

*Report of the Pennsylvania Hospital for the Insane, for the year* 1847. By Thomas S. Kirkbride, M. D., Physician to the Institution. Philadelphia, 1848.

In no other branch of the healing art have greater improvements been made within the last quarter of a century than in that which concerns the treatment of the Insane. Formerly, the places provided for these unfortunates were prisons, often of the worst kind, where the unhappy inmates were treated with even less of kindness and respect than felons. Beside the shocking influence of such treatment upon the feelings of the well, nothing could be more prejudicial in its effects on the minds of the sufferers, than the constant impression of the insults, neglect, and, in many cases, wanton outrages, to which they were subjected. Science and philosophy have, however, led to a system in better accordance with the dictates of humanity.

In looking over the reports which annually emanate from the institutions devoted to the relief of the Insane, one is delighted to observe the steady progress which is made in the moral and physical management of this class of patients; how, step by step, from being regarded as beyond the pale of humanity, they have become the especial objects of sympathy, and made to participate in the rational enjoyments of life, even while labouring under that greatest of all deprivations. Dr. Earle is distinguished for the zeal and ability with which he has laboured in this interesting field, and the publication cited above, for which we are indebted to him, affords evidence on almost every page of his rare qualifications for the task. A history of an Insane Asylum would seem to afford little for entertainment or agreeable reflection, and yet we have found such publications of latter time among the most agreeable books to read.

The history of the Bloomingdale Asylum is divided by the author into three *Parts.*

*Part first* treats of the "*Origin and progress of the Bloomingdale Asylum,* and of *the Reception of Patients,*" and is interesting from the incidents connected with its early history as a branch of the New York Hospital.

*Part second* is devoted to an account of the "*present state of the Asylum,*" and it is in this that we find the information which concerns us most; as the *moral treatment* of the patients, including the subjects of Manual Labour, Religious Worship, and Recreative Exercise; *Instruction, Amusements, Restraints,* etc. The following remarks on the *moral treatment* pursued, express the views of the most enlightened men of the present day, whose minds are devoted to this subject.

"In the moral regimen at this institution, every practicable effort is made to pursue that system, at once gentle, philosophical and practical, which has resulted from the active and strenuous endeavors of many philanthropists, in the course of the last half century, to meliorate the condition of the insane. The primary object is to treat the patients, so far as their condition will possibly admit, as if they were still in the enjoyment of the healthy exercise of their mental faculties. An important desideratum for the attainment of this object is, to make their condition, as boarders, as comfortable as possible; that they may be the less sensible of the deprivations to which they are subjected by a removal from home. Nor is it less essential to extend to them the privilege, or the right, of as much liberty, as much freedom from personal restraint as is compatible with their safety, the safety of others, and the judicious administration of other branches of curative treatment. The courtesies of civilized and social life are not to be forgotten, tending, as they do, to the promotion of the first great object already mentioned, and operating, to no inconsiderable extent, as a means of effecting restoration to mental health."

"*Manual Labour.*—Some employment for the hands, of a description requiring a degree of exercise of the body sufficient to preserve and increase the activity and vigor of all its organs, as well as to promote sound and healthful sleep, is acknowledged, by all who are conversant with the treatment of insanity, as it appears in public institutions, to be the most effectual of restorative measures not purely medical. Hence, some physicians have recommended compulsory labour, in cases where the patient will not engage in it voluntarily.

At this Asylum the patients are advised and, if possible, induced to apply themselves to some useful occupation, but no compulsory measures are resorted to for the purpose of enforcing it.

A large proportion of the inmates, as will be perceived by the table of occupations, are from the classes unaccustomed to manual labour. These, with very rare exceptions, will never commence any employment of the kind, while at the Asylum.

Another large class of the patients are mechanics and artisans, from the city, unaccustomed to any occupation other than that to which they were bred, and facilities for pursuing which cannot, in most cases, be furnished here or at any similar institution.

Finally, of those who are acquainted with, and habituated to such kind of labour as can be introduced here, a considerable proportion are rendered unfit to work by their disease; and others, though able, will not work, because—to them apparently the best of all reasons,—they 'pay their board.'

With all these adverse influences, it cannot be expected that, in an institution accommodating but about seventy-five of either sex, the number should be large who devote much of their time to labour. There are some, however, who work upon the farm, and others in the carpenter's shop, the kitchen and the laundry; while numerous small jobs about the establishment furnish employment to a considerable number of the men.

A much larger proportion of women than of men labour voluntarily, not only because all classes of women, in this country, are, with but few exceptions, taught to use the needle, but because this species of labour is one which can be introduced into the apartments of the patients."

There is much in this valuable *brochure* worthy of quotation, if our space would permit.

The Report of the Pennsylvania institution, as on former occasions, is a highly interesting publication. Located in our immediate neighbourhood, we have the opportunity of witnessing its progress, and appreciating the excellent management under the fostering influence of which its benefits are annually increasing.

"Since the date of the last Report," says Dr. Kirkbride, "240 patients have been admitted, 213 have been discharged or died, and 188 remain under care at the close of the year."

"The total number under care during the year has been 401, the average number during the year has been 185 nearly, and as many as 201 have been in the hospital at one time."

Notwithstanding the additions made to the buildings of the north wing, during the preceding year, every room in the Hospital has been filled for several weeks continuously, and some difficulty has been experienced in accommodating all who were brought to the institution. By considerable effort, however, no proper applicant has been refused, and the Hospital has been made as useful as its extensive buildings would permit.

It is a source of gratification to be able again to report, what could not have been anticipated, that from the opening of the institution, with a solitary exception in one item, each year has witnessed a steady increase in the number of admissions, in the total number of patients during the year, in the number in the house at one time, and in the number of recoveries.

In the past year, this has been more striking than any which preceded it; the number of admissions having been 73 more than in the previous year; the number of recoveries 22 more; the average number under care during the whole year has risen from 173 to 185; and the number at the close of the year is 27 more than last reported. Without the additions made to our buildings, none of these results could have been effected.

The new structures added to the North Lodge, and just completed at the date of the last Report, were soon after furnished and opened for the reception of patients. In less than three months from that time, every room in them was occupied, and even that prepared for the seamstress and female nurse, has been claimed and used by a patient with a private attendant.

A year's experience has demonstrated to the satisfaction of every one, the immense importance of these improvements in the classification of the patients, in the comfort and welfare of all the inmates, and that their erection could not have been longer delayed, without serious loss and disadvantage to the institution."

The same moral influences are invoked for the restoration of patients in this as in the preceding and other valuable institutions of a kindred character in the United States.

"*Employment and Amusements.*—The frequent inquiries made in reference to the means of employment and amusement in use in this institution, render it proper to refer to the subject somewhat more in detail than has been customary in the reports of the last few years.

The proper mental and physical employment of the insane is of so much importance, and embraces such a great variety of means, that a review of the whole subject, with all the details of arrangements to effect the object, would be nearly equivalent to giving a complete essay on the moral treatment of insanity. Without attempting to do this, I shall refer to those means which are constantly in requisition, and in reference to a few, add some remarks which have been suggested by experience.

Let the employment be what it may, if possible, a degree of interest should be excited in the patients; there should be utility combined with it, and, in most cases, physical with mental occupation is desirable. Active exercise in the open air, moderate labor in the garden, about the pleasure grounds, the flower borders, or on the farm, stand prominent for good results. In the work-shop, occupation in carpentering, joining, mattress making, turning and making fancy or useful articles, is available to many. Walking inside of the enclosure is intended to be used by nearly every patient in the house, twice a-day, during the entire year, except in stormy weather, and the paved, gravel, or tan walks, between two and three miles in extent, offer facilities for the purpose. The green-house and care of plants please some in the proper season, while in hot weather many spend most of their time in the open air, in the groves and woods, which are neatly fitted up with summer houses, rustic seats, &c.

A considerable number indulge in long walks outside of the inclosure, either to the many objects of interest in the city of Philadelphia, or to attractive spots even many miles from the institution. Short excursions are occasionally made on railroads or in steamboats. The carriage and horses are in constant use. Exercising swings, the pleasure railroad, the ten-pin alley, and various other active games in the open air, are at times, enjoyed by many.

Within doors, are many more decidedly mental means of occupation. Among these may be mentioned the use of a good library of about eleven hundred volumes, a great variety of newspapers and periodicals, conversation, writing, the study of some of the languages, mathematics, or other favorite branches of science; reading aloud to the patients of certain wards, by the teachers or attendants, the regular course of lectures already referred to, exhibitions of a great variety of interesting subjects by means of the magic lantern, very beautiful dissolving views, boxes for perspective views, a large assortment of engravings, prints, and illustrated papers, and the examination of curiosities and specimens of natural history. Various musical instruments are used by individual patients, and we have occasional concerts with social parties. We have nearly every variety of games for those who take an interest in them, and every form of innocent employment, which taste, utility, or previous pursuits may indicate, is encouraged. Many varieties of fancy work are favorites with the patients, and a large number derive great advantage from the cheerful aid they offer in assisting the attendants and others in the performance of their various duties about the house."

